# Interrupted Inferior Vena Cava Combined with Partial Anomalous Pulmonary Venous Return Drainage to the IVC in a 67-Year-Old Adult

**DOI:** 10.1111/jocs.12039

**Published:** 2012-12-10

**Authors:** Hee Jae Jun

**Affiliations:** Department of Thoracic and Cardiovascular Surgery, Haeundae Paik Hospital, Inje UniversityBusan, Republic of Korea

## Abstract

A 67-year-old woman presented with lower body edema and was found to have a suprarenal inferior vena cava (IVC) obstruction without hepatic vein obstruction and partial anomalous pulmonary venous return (PAPVR) draining the right pulmonary veins to the IVC below the obstructed IVC on CT angiography. The patient underwent retrohepatic cavoatrial bypass with a polytetrafluoroethylene (PTFE) 16-mm ringed graft via a posterolateral thoracotomy and retroperitoneal approach.

The combined anomaly of a suprarenal inferior vena cava (IVC) obstruction without hepatic vein obstruction and a partial anomalous pulmonary venous return (PAPVR) is an extremely rare condition. We present a case with this complex congenital anatomy and describe its operative management.

## CASE REPORT

**Figure 1:**
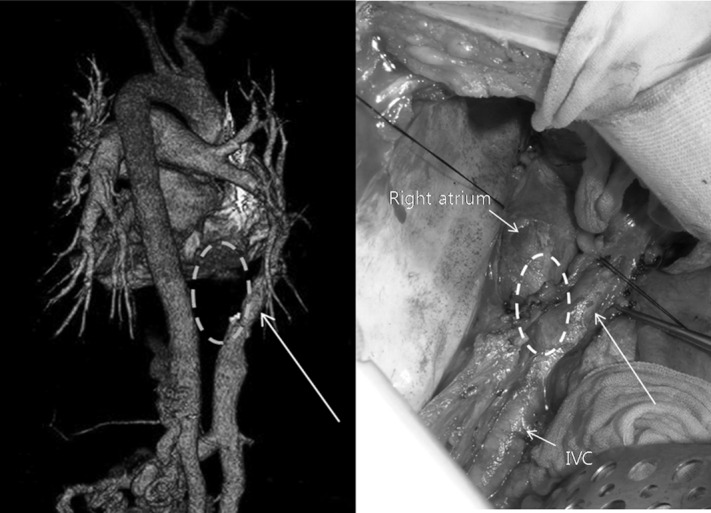
A reconstructed computed tomography (CT) angiography and intraoperative findings showed interruption of IVC (white circle), and anomalous right pulmonary vein (white arrow) drained into the IVC.

**Figure 2:**
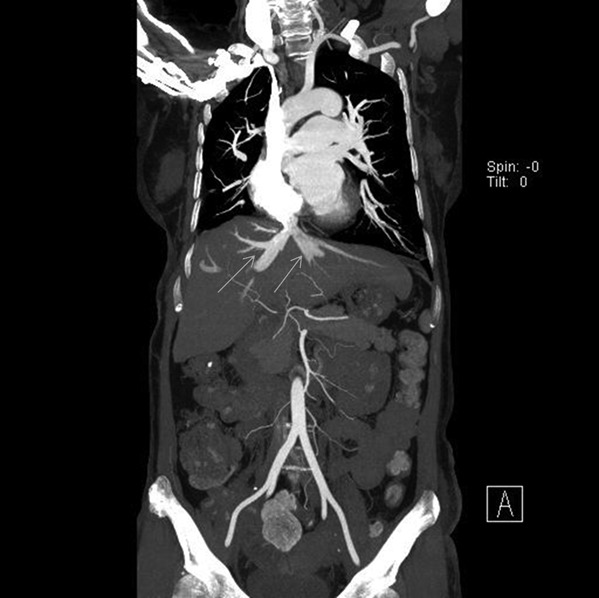
Computed tomography (CT) coronal view demonstrated the hepatic veins draining directly into the right atrium (red arrow).

**Figure 3:**
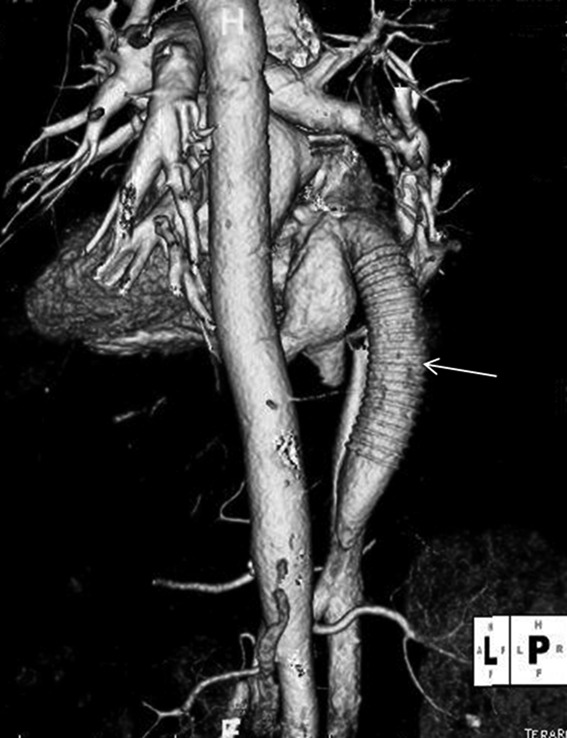
Postoperative reconstructed CT image shows the ringed PTFE graft (arrow) from suprarenal IVS to right atrium with good blood flow.

A 67-year-old female presented with chronic edema of the lower abdomen, perineum, and bilateral lower extremities. Renal function was abnormal (BUN/creatinine 22/1.52). Echocardiography showed normal function of both ventricles, however the right-sided pulmonary veins were not identified. The systolic pulmonary arterial pressure was 34 mmHg. Computerized tomography (CT) angiography showed total interruption of the IVC below the hepatic veins and confirmed the abnormal return of the right-side pulmonary veins into the subdiaphragmatic IVC below an IVC obstruction (Fig. 1). There were IVC collaterals to the azygos vein draining into the superior vena cava and hepatic veins draining directly into the right atrium (Fig. 2). At the time of surgery, a posterior lateral thoracotomy incision was made, and the chest was opened through the ninth intercostal space. The eighth and ninth ribs were divided posteriorly. Division of the diaphragm was extended medially to the hiatus of the IVC. The right side of the retrohepatic IVC was exposed by retroperitoneal dissection (Fig. 1). The patient underwent retrohepatic cavoatrial bypass from right atrium to the suprarenal IVC with a PTFE 16-mm ringed graft (Gore-Tex, W. L. Gore & Associates, Inc., Flagstaff, AZ, USA; Fig. 3). The preoperative CVP and IVC pressure were 8 and 13 mmHg and the postoperative IVC pressure was 8 mmHg. The patient recovered without any complications. Her symptoms were resolved. The graft has remained patent on images obtained two years following the surgery. The patient has taken warfarin and aspirin to maximize the graft patency.

## DISCUSSION

Failure of hepatic and prerenal segments fusion is the most common developmental anomaly of the IVC and results in infrahepatic IVC interruption. Infrahepatic IVC interruption with azygos continuation is a rare congenital anomaly. Its prevalence is 0.6% to 2.0% in patients with congenital heart disease and less than 0.3% among otherwise normal patients.^1^ The infrahepatic IVC may continue as the azygos vein or the hemiazygos vein, and drains to the left superior vena cava, intrathoracic veins, or anomalous intrahepatic veins. The hepatic segment of the IVC drains directly into the right atrium. The IVC interruption may be associated with recurrent deep vein thrombosis of the lower limbs and bilateral venous insufficiency. There can be procedural difficulties during right heart catheterization, cardiopulmonary bypass surgery, femoral vein catheter advancement, IVC filter placement, and temporary pacing through the transfemoral route.

Dupuis et al.^2^ showed that in patients with PAPVR, a left-to-right shunt is present in fewer than 50% of patients with slightly elevated pulmonary artery pressures. These patients were able to lead a normal life without surgical correction. In our case, conservative treatment was preferable to surgical correction of PAPVR because this patient did not have any heart failure symptoms and the systolic pulmonary artery pressure was only 34 mmHg. Surgical correction of PAPVR involves: (1) creating a long baffle from the orifice of the scimitar vein within the IVC to the atrial septal defect,^3^ (2) division with reimplantation of the scimitar vein into the right atrium with an intra-atrial baffle,^4^ and (3) direct anastomosis of the divided scimitar vein to the left atrium.^5^ In this case, Baffle surgery was impossible because of the IVC interruption. We decided that direct anastomosis of the anomalous pulmonary vein and left atrium would be a dangerous approach because this patient's heart was dextro-rotated. A direct anastomosis may have caused obstruction of the anastomosis because of in-folding and kinking of the vein (Fig. 1).
